# Evaluation of the Prevalence of Exons 1 And 10 Polymorphisms of *LHCGR* Gene and Its Relationship with IVF Success

**Published:** 2019

**Authors:** Maliheh Javadi-Arjmand, Elia Damavandi, Hamid Choobineh, Fereshteh Sarafrazi-Esfandabadi, Majid Kabuli, Atoosa Mahdavi, Mohsen Ghadami

**Affiliations:** 1- Department of Medical Genetics, School of Medicine, Tehran University of Medical Sciences, Tehran, Iran; 2- Specialized Medical Genetic Center of Academic Center for Education, Culture and Research (ACECR), Tehran University of Medical Sciences Branch, Tehran, Iran; 3- Department of Photo Healing and Regeneration, Medical Laser Research Center, Yara Institute, ACECR, Tehran, Iran; 4- School of Allied Medicine, Tehran University of Medical Sciences, Tehran, Iran; 5- Department of Obstetrics and Gynecology, Dr. Shariati Hospital, Tehran University of Medical Sciences, Tehran, Iran; 6- Endocrinology and Metabolism Research Institute, Tehran University of Medical Sciences, Tehran, Iran; 7- Cardiac Primary Prevention Research Center, Tehran Heart Center, Tehran University of Medical Sciences, Tehran, Iran

**Keywords:** *In vitro* fertilization, Infertility, *LHCGR* gene, Polymorphism

## Abstract

**Background:**

Luteinizing hormone receptor gene shows four nonsynonymous polymorphisms within the exons. Three of these polymorphisms, *i.e*. rs4539842 (an insertion of 6bp CTGCAG at nucleotide position 54), rs12470652 (c.827A>G/p.Asn 291Ser), and rs2293275(c.935G>A/p.Ser312Asn) have been studied more frequently. Beside other hormones, LH and FSH have an important role in production of competent oocyte and female fertility. Therefore, the objective of the current study was to investigate the prevalence of exons 1(rs4539842) and 10(rs12470652, rs2293275) polymorphisms of the *LHCGR* gene and its relationship with successful IVF in Iranian infertile women.

**Methods:**

SNPs in exons 1 and 10 were analyzed in 100 women of two equally sized groups of IVF failure and IVF success women using genomic DNA. For polymorphisms in exon 10, PCR and direct sequencing were used and for the polymorphism in exon 1, RFLP technique was used. The RFLP technique is confirmed by sequencing.

**Results:**

Our results showed significant difference in allelic frequency of SNP rs2293275 among IVF successful and IVF failure groups (p=0.001). For this variation, AA genotype (A allele) was shown to have protective effect against IVF failure (p=0.03 and OR=0.04), while GG genotype (G allele) was a susceptive genotype to IVF failure (p=0.003 and OR=3.88).Allelic frequency of SNP rs4539842 also showed significant difference between the two groups (p=0.0025). For this SNP, subjects with no 6bp insertion (homozygote deletion genotype) were susceptible to failure in IVF (p=0.009 and OR=2.93).

**Conclusion:**

It has been revealed that two common SNPs (rs4539842 and rs2293275) in the *LHCGR* gene are associated with the outcome of IVF in Iranian infertile women. Thus, these two SNPs can be suggested to be used as predictors for IVF outcome in Iranian population.

## Introduction

Infertility is characterized by the failure to establish clinical pregnancy after 12 months of regular unprotected sexual intercourse, and is estimated to affect 8–12% of couples of the reproductive age worldwide (1). The primary infertility rate in Iran is 20.2% (2). A combination of both genetic and environmental factors leads to infertility. Knowledge of the exact causes of infertility in couples leads to adoption of the best and most effective therapeutic procedures. *In vitro* fertilization (IVF), the most famous assisted reproduction method, has been developed and improved to benefit millions of childless couples (3). This costly method involves extracting a woman’s eggs, fertilizing the eggs in the laboratory, and then transferring the resulting embryo(s) into the woman’s uterus (4). The financial and emotional costs associated with IVF failure can be devastating for infertile couples. Therefore, finding the causes of IVF failure and factors leading to successful IVF is very important for infertile couples. Luteinizing hormone is essential for growth and maturity of sexual organs and secondary sexual characteristics. In women, an acute LH rise triggers ovulation and corpus luteum development. Novel experimental and clinical evidence suggests that administration of pharmacological doses of LH, instead of being harmful, is therapeutically advantageous, particularly in supporting and modulating ovarian folliculogenesis (5). Although ovarian stimulation is efficiently achieved in most cases by administration of exogenous FSH alone, specific subgroups of women may benefit from LH activity supplementation during ovarian stimulation (6). It has been shown that the use of LH along with FSH improves the fertilization rate, implantation rate, pregnancy rate, and live birth rate in patients undergoing ovarian stimulation in IVF cycles (7). The luteinizing hormone receptor (LHCGR) is a classical G-protein coupled receptor predominantly expressed on theca cells of ovary, but it is also expressed on granulosa and cumulus cells (8–10). The theca, stromal, late-stage (luteinizing) granulosa, and luteal cells in ovary and testes contain LHCGR (11). The *LHCGR* gene located on the chromosome 2p21, contains 11 exons and spans approximately 80 *kb* (12, 13). The gene was first sequenced in 1990 (14), and more than 300 known polymorphisms have been described for this gene; however, only four are nonsynonymous and occur within exons. Exon 10 of the gene is important for receptor activation by LH through a mechanism involving extracellular conformational changes (15). The four nonsynonymous polymorphisms in the exons of *LHCGR* are rs12470652 (c.827A>G/p.Asn 291Ser), rs2293275 (c.935G>A/p.Ser312Asn), rs 4539842 (insLQ) and rs13006488 in exon 11 (16). An insertion of two amino acids (leucine [L] and glutamine [Q]) in the signal peptide of the LHCGR (18insLQ) has been shown to result in increased receptor activity *in vitro* (17). It has been recently shown that the *LHCGR* variant N312S and the *FSHR* variant N680S can be used for prediction of pregnancy in women undergoing IVF (18). A cross-sectional study conducted by Lindgren et al. showed that women homozygous for serine (S) in the follicle-stimulating hormone receptor (*FSHR*) variant N680S and the luteinizing hormone/human chorionic gonadotrophin receptor (*LHCGR*) variant N312S displayed higher pregnancy rates after IVF procedure than women homozygous for asparagine (N). Increased pregnancy rates were also evident for women carrying *LHCGR* S312, regardless of the *FSHR* variant. These women required higher doses of FSH for follicle recruitment than women homozygous for N (18). In this study, three polymorphisms of the *LHCGR* gene, including rs4539842, rs12470652, and rs2293275, and their relationship with successful IVF in Iranian women were examined.

## Methods

### Subjects:

This case-control study was performed in patients attending the infertility clinic at Dr. Shariati Hospital, Tehran, Iran. After obtaining informed consent from all participants, blood samples were collected from 50 infertile women with unsuccessful IVF and 50 infertile women with successful IVF.

Our criterion for successful IVF was clinical pregnancy confirmation based on the blood test of participants and observation of gestational sac and embryonic heart activity after treatment with IVF. The IVF failure group were participants for whom clinical pregnancy was not confirmed after two IVF cycles. Inclusion criteria were a regular menstruation cycle of 21–35 days, age below 40 years old, and lack of smoking. Moreover, the reason for IVF in couples was not due to male factor such as azoospermia and varicocele. The patients did not have known causes of infertility, such as PCOS or endometriosis. The basal and clinical characteristics of successful and unsuccessful IVF groups has been shown in Table 1. The study received approval from the ethics committee of Tehran University of Medical Sciences.

**Table 1.** Basal and clinical characteristics of successful and unsuccessful IVF groups

**Variables**	**Successful IVF**	**Unsuccessful IVF**
**Age (year)**	28.22±4.73	31.62±4.74
**Body mass index (*kg/m*^2^)**	25.35±3.85	24.85±4.32
**Marriage duration (year)**	7.11±3.37	7.5±4.5
**Infertility duration (year)**	5.11±3.37	5.5±4.3
**Anti-mullerian hormone (*ng/ml*)**	7.86±5.02	5.9±6.58
**Luteinizing hormone (*mIU/ml*)**	5.52±2.27	6.32±2.01
**Follicle-stimulating hormone (*mIU/ml*)**	5.57±3.14	4.58±1.82
**Estradiol (*pg/ml*)**	45.08±20.51	39.15±33.12
**Antral follicle count**	12.3±2.75	6.2±2.48
**Stimulation days**	12.7±1.97	12.8±1.64
**Transferred embryos**	2.4±0.69	2.7±0.48

Data shown as M±SD. Follicle-stimulating hormone, luteinizing hormone and estradiol were measured on day 3 of the cycle.

### Patient treatment:

After complete desensitization (long-term protocol) using the gonadotrophin-releasing hormone agonist, buserelin (Daroo Sazi Samen Co., Iran), ovarian stimulation with Gonal F (Serono, Switzerland) was started on the third day of the next cycle. The progression of follicle development was monitored by transvaginal ultrasound every 3–5 days. After observation of at least two follicles, human chorionic gonadotropin (hCG; Ferring Co., Germany) was administered and 36–38 *hr* later, oocyte retrieval was performed.

### DNA extraction:

From each participant, 5 *ml* blood was collected in EDTA-containing tubes. Genomic DNA extraction was done using the QIAamp DNA Mini Kit (QIAGEN) and Phenol-Chloroform method.

### Analysis of polymorphism rs4539842 in exon 1:

PCR amplification of exon 1 of the *LHCGR* gene was conducted with the following primers according to Atger et al.

Forward primer: 5′-CACTCAGAGGCCGTCCAAG-3′ Reverse primer: 5′-GGAGGGAAGGTGGCATAGAG-3′.

Each PCR reaction contained 1 *μl* genomic DNA (100–200 *ng*), 1 *μl* of each primer (with concentration 10 *pmol/μl*), 2 *μl* DMSO 20% and 12 *μl* Master Mix. The aliquot was increased to a final volume of 25 *μl* by adding 8 *μl* distilled water. Amplification was performed under the following conditions: first denaturation at 95*°C* for 5 *min* followed by 30 cycles of second denaturation at 95*°C* for 30 *s*, annealing at 65*°C* for 30 *s*, extension at 72*°C* for 20 *s*, and final extension at 72*°C* for 5 *min*. To confirm specific amplification, amplicons were submitted to electrophoresis on the 1% agarose gel. Amplicons were then digested with *PVUII* and electrophoresed on the 3.5% agarose gel. Direct DNA sequencing was done to confirm the results of RFLP.

### Analysis of polymorphisms rs12470652 and rs 2293275 in exon 10:

The Gene Runner software and Oligo Explorer 1.5 were used to design primers for exon 10 of the *LHCGR* gene. The primer sequences were 5′-TGGAAAGGAGAGTGGTAAGG-3′ for forward primer and 5′-GAAGGTATCAGTTGTATCAGCC-3′ for reverse primer, which amplified a 744-*bp* product size. Polymorphisms in exon 10 of the *LHCGR* gene were identified by PCR sequencing. Each PCR reaction contained 1 *μl* genomic DNA (100–200 *ng*), 1 *μl* of each primer (with concentration 10 *pmol/μl*), 2 *μl* DMSO 20%, and 12 *μl* Master Mix. The aliquot was increased to a final volume of 25 *μl* by adding 8 *μl* distilled water. Amplification was performed under the following conditions: first denaturation at 95*°C* for 5 *min* followed by 30 cycles of second denaturation at 95*°C* for 30 *s*, annealing at 60*°C* for 30 *s*, extension at 72*°C* for 20 s*,* and final extension at 72*°C* for 5 *min*. To confirm the specific amplification, amplicons were submitted to electrophoresis on the 1% agarose gel. PCR products were sequenced with the ABI-3130 automated sequencer.

### Statistical analysis:

Data were analyzed using SPSS statistical software (IBM Corp. Released 2016. IBM SPSS Statistics for Windows, Version 24.0. Armonk, NY: IBM Corp.) p<0.05 was considered statistically significant. Significance of differences between groups was assessed by the Chi-square test.

## Results

**Figure 1.**
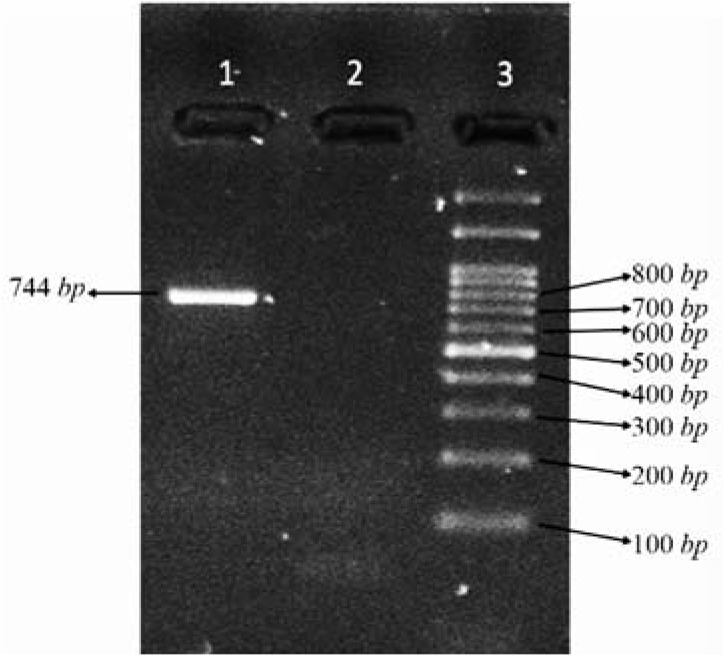
Electrophoresis of the PCR product of exon 10 (Lane 1: PCR product, Lane 2: no template control (NTC), Lane 3: 100 *bp* DNA ladder)

**Figure 2.**
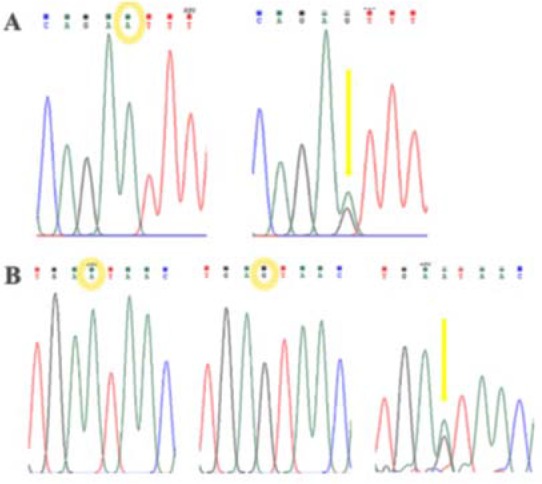
A: The graph of sequencing results for rs12470652. B: The graph of sequencing results for rs2293275

**Figure 3.**
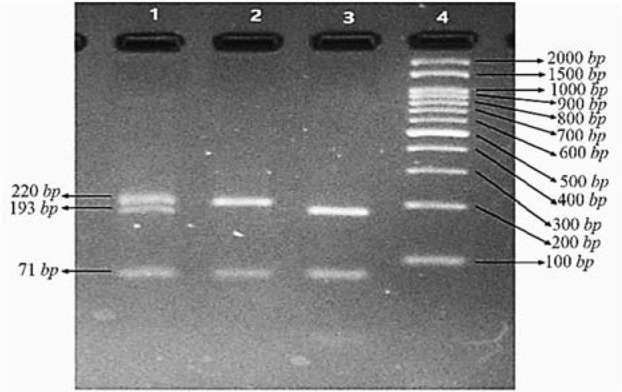
Electrophoresis of three samples after digestion with *PVUII*. (Lane 1: heterozygote for the 6*bp* insertion, Lane 2: no insertion, Lane 3: homozygote for the 6*bp* insertion, Lane 4: 100 *bp* DNA ladder)

**Figure 4.**
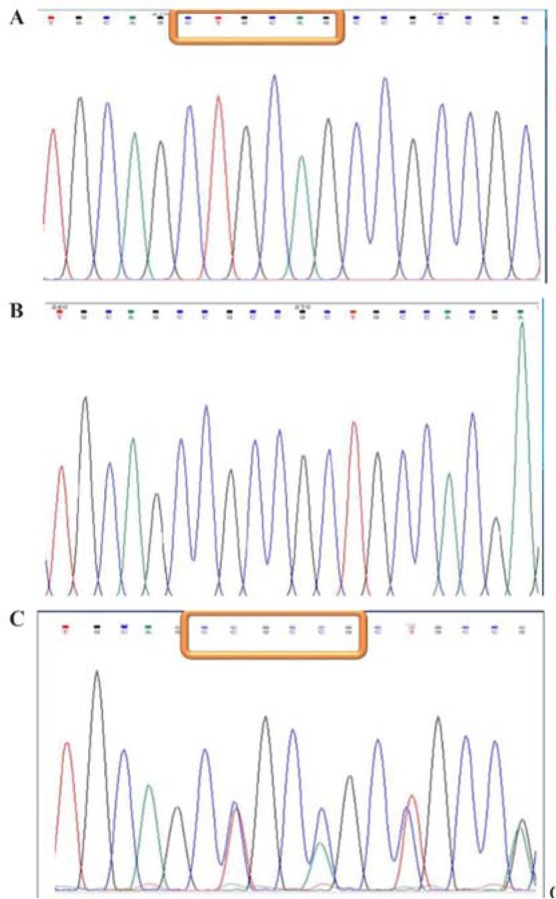
Direct sequencing of exon 1. A: homozygote for the 6bp insertion, B: homozygote without the insertion, C: heterozygote for the 6bp insertion

To identify SNPs in exon 1 and 10 of the human *LHCGR* gene, genomic DNA was extracted and these regions were amplified by PCR. Amplicons of exon 10 were attained by agarose gel electrophoresis (Figure 1) and sequenced to find SNPs in exon 10 (Figure 2). For exon 1, amplicons were digested with *PVUII* and subjected to electrophoresis on the 3.5% agarose gel (Figure 3). Direct DNA sequencing was performed to confirm the results of RFLP (Figure 4).

The distribution of allelic and genotypic frequencies between successful IVF and unsuccessful IVF subjects for each SNP has been shown in table 2. The comparison of expected allelic and genotypic frequencies with observed frequencies showed that the studied population is in Hardy-Weinberg equilibrium for the three SNPs.

**Table 2.** Allelic frequencies and the genotype distribution of the SNPs rs2293275 and rs4539842 in the successful and unsuccessful IVF groups

**SNP**	**Alleles/genotypes**	**Unsuccessful IVF (n=50)**	**Successful IVF (n=50)**	**p-value**	**OR(95% CI)**
**Rs2293275**					
	A	27 (27%)	50 (50%)		0.37(0.2–0.67)
	G	73 (73%)	50 (50%)	0.001	2.7(1.5–4.8)
	AA	0 (0%)	9 (18%)	0.03	0.04(0.002–0.76)
	AG	27 (54%)	32 (64%)	0.31	0.66(0.29–1.47)
	GG	23 (46%)	9 (18%)	0.003	3.88(1.56–9.65)
**Rs4539842**					
	INS	16 (16%)	35 (35%)		2.83 (1.44–5.55)
	DEL	84 (84%)	65 (65%)	0.0025	0.35(0.18–0.69)
	Homozygote insertion	0 (0%)	6 (12%)	0.07	0.068(0.004–1.24)
	Heterozygote	16 (32%)	23 (46%)	0.153	0.55(0.24–1.25)
	Homozygote deletion	34 (68%)	21 (42%)	0.009	2.93(1.3–6.65)

A statistically significant difference was found between successful IVF and unsuccessful IVF subjects in the allelic frequencies of rs2293275 (p= 0.001). For this variation, our finding indicated that the GG genotype makes women susceptible to failure in IVF (OR=3.88, 95% CI=1.56–9.65, p= 0.003). However, the AA genotype had protective effect (OR=0.04, 95% CI=0.002–0.76, p=0.03). Carrier genotype of AG had no effect on predisposing women to failure in IVF (OR=0.66, 95% CI= 0.29–1.47, p=0.31). For second SNP (rs12470652), no significant difference was found between successful IVF and unsuccessful IVF subjects in the allelic frequencies (p=0.099). However, for the SNP located in exon 1 (rs4539842), the allelic distribution differed significantly between successful IVF and unsuccessful IVF in women (p=0.0025). This study showed that homozygote women with no insertion of rs4539842 genotype are susceptible to failure in IVF (OR=2.93, 95% CI=1.3–6.65, p= 0.0099). But for the heterozygote genotype and homozygote insertion genotype, no association was found regarding the outcome of IVF. For the SNP rs12470652, no significant difference was found in allelic frequency among IVF successful and IVF unsuccessful subjects (p= 0.09).

## Discussion

The incidence of infertility is estimated to be 8–12% worldwide and is slightly more frequent in Iran with the rate of 20.2% for primary infertility (2). Various factors including genetic and environmental factors are known to have a role in infertility. Among these factors, the *LHCGR* gene and its polymorphisms have been less studied in different populations, including the Iranian population. The normal function of the *LHCGR* gene is essential for successful fertility in both sexes. It has been shown that targeted disruption of the *LHCGR* gene in embryonic stem cells results in infertility in both sexes (19). Two groups of investigators independently reported the development of *LHCGR* knockout mice by deleting part of the promoter region and exon 1 (19) and 11 encoding the transmembrane and intracellular domain of the receptor (20). Both models showed a complete loss of functional receptor resulting in infertility in both sexes. Therefore, the large number of naturally occurring mutations and polymorphisms in the *LHCGR* gene that result in disorders of sexual development and reproductive function highlights the critical role of this receptor in reproduction. Regarding the *LHCGR* gene polymorphisms and their associations with other diseases, different studies have shown that rs2293275 is associated with polycystic ovarian syndrome (21–23). Moreover, the *LHCGR* 312Asn allele can be regarded as a weak breast cancer risk allele. However, no associations have been found between breast cancer and the *LHCGR* 291Ser allele but it is associated with increased receptor sensitivity (24). The rs4539842 polymorphism is also associated with a shorter disease-free survival in breast cancer patients (17, 25).

Considering polymorphisms in the *LHCGR* gene and the probability of pregnancy and the success of IVF among women, it is necessary to study these polymorphisms in Iranian women. The present study examined three polymorphisms in the *LHCGR* gene (rs4539842, rs12470652 and rs2293275) and their association with successful IVF. The results demonstrated a significant difference (p<0.05) in the genotype distribution frequency among the two groups and IVF outcomes for rs4539842 and rs 2293275. In the present study, a strong association was found between SNP rs2293275 in *LHCGR* and successful IVF. Frequency of G allele was statistically higher in the IVF failure group compared with IVF successful group; or in other words this allele is a risk factor for unsuccessful IVF. In this regard, it has been found that GG genotype is a susceptibility factor for IVF failure, while AA genotype has a protective effect against IVF failure. Regarding the SNP rs4539842, a strong association was found between this SNP and successful IVF. Since no insertion (del) allelic frequency was apparently higher in IVF failure subjects than IVF successful subjects, having no insertion of 6*bp* CTGCAG in women can be regarded as a risk factor. Subjects who do not have the 6*bp* insertion (homozygote deletion) showed to have higher risk for unsuccessful IVF in comparison with heterozygote and homozygote insertion genotype.

## Conclusion

Controversial findings in different populations regarding the effect of *LHCGR* polymorphisms on infertility and IVF success suggest the need for more investigation at a molecular level and in other populations. Furthermore, combined assessments including *LHCGR* and *FSHR* polymorphisms should be done in different populations, including the Iranian population. However, to confirm the results of the present study, a larger number of Iranian infertile women with successful and unsuccessful IVF should be examined. Thus, the additive effect of such genes must be studied to uncover possible genetic causes of infertility in detail and formulate personalized treatments for infertile women.
